# INTRA-INDIVIDUAL PHYSICAL AND PSYCHOLOGICAL SYMPTOM VARIABILITY AND STRESS INFLUENCE ACTIVITIES OF DAILY LIVING

**DOI:** 10.1093/geroni/igz038.993

**Published:** 2019-11-08

**Authors:** Scott E Moore, Kelly Wierenga, Heather K Hardin

**Affiliations:** 1 Frances Payne Bolton School of Nursing, Case Western Reserve University, Cleveland, Ohio, United States; 2 Indiana University, Indianapolis, Indiana, United States

## Abstract

Stress and symptomatology have been shown to have strong relationships to health outcomes in older adults. More specifically, perceived social stressors (PSS), whether related to disease, disability or demographics, is a contributor to health. Recently it was reported that intra-individual symptom variability (ISV) may predict poor health outcomes better than symptom severity in some chronic illnesses. Individual and combined influences of ISV and PSS on health behaviors are not fully described. Using a subset (n = 518, 46.5% men; mean age = 48.7) of MIDUS Refresher participants’ 8 day daily diary data, we sought to determine the influences of physical and psychological ISV and PSS on independent and basic activities of daily living (iADLs, bADLs). The ISVs represent an average of day-to-day variation across each of the 22 physical and 27 psychological symptoms for each participant. Psychological ISV, physical ISV, PSS, and total number of chronic conditions were entered into two structural equation models as predictors for each ADL outcome (p<.01). The models depicted both direct and indirect influences of psychological ISV on ADLs (iADLs: B=-.43, P < .001; B = .51, P < .001 [through PSS]; bADLs: B=-.45, P < .001; B = .51, P < .001 [through PSS]). However, the influence of physical ISV on ADLs was indirect (B = .22, P = .001 [through PSS]). Individual-level influences of ISV and PSS on ADLs may better aide healthcare providers’ identifying and intervening to disrupt poor health outcomes for those at risk.

